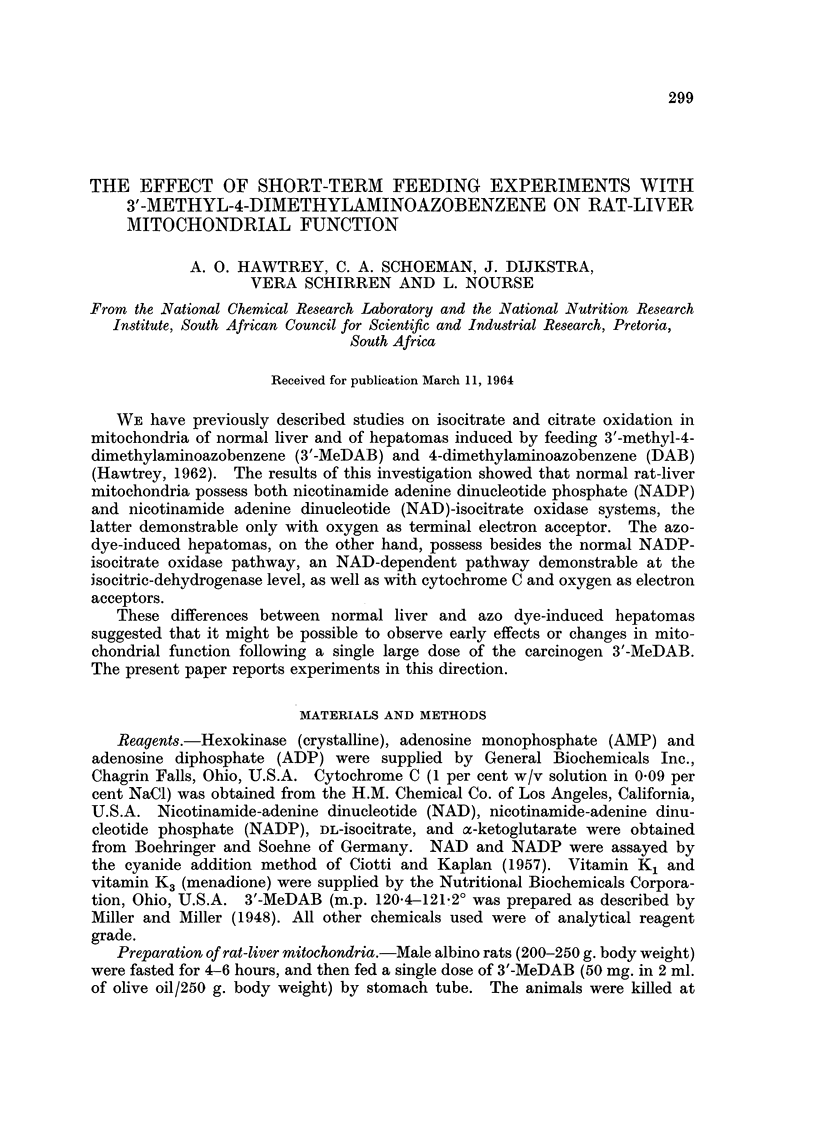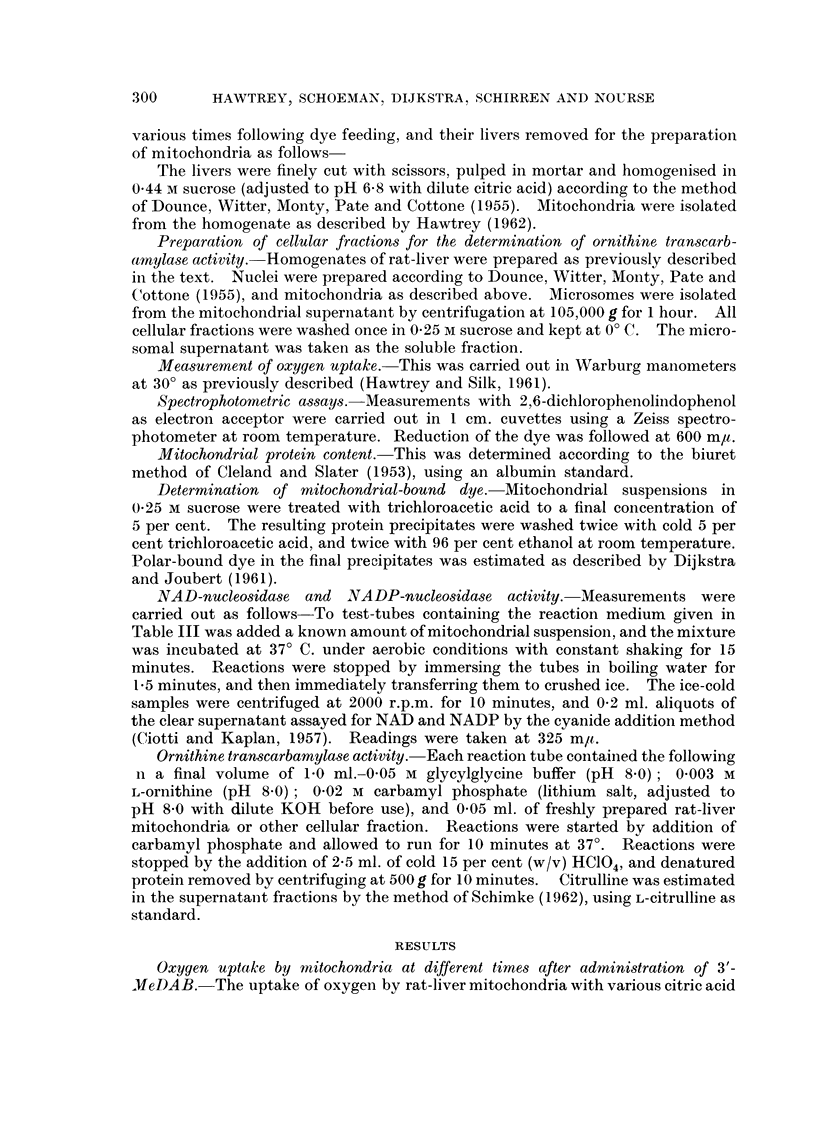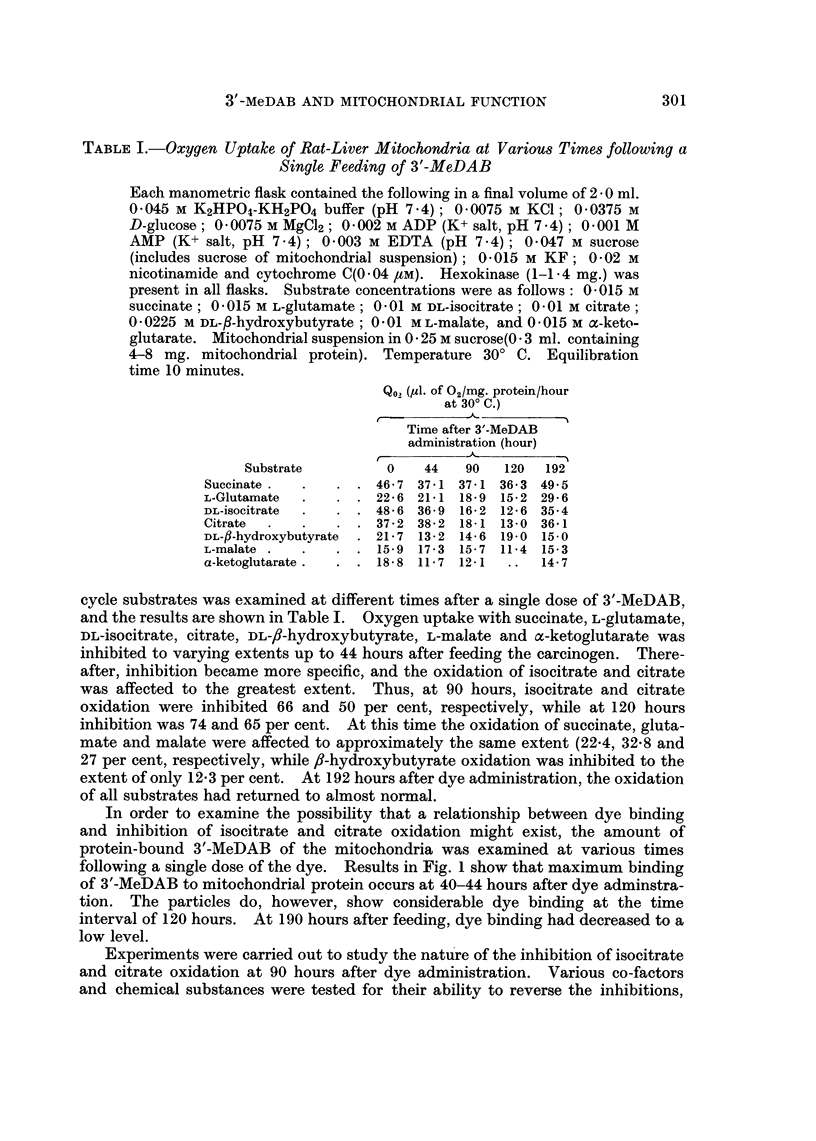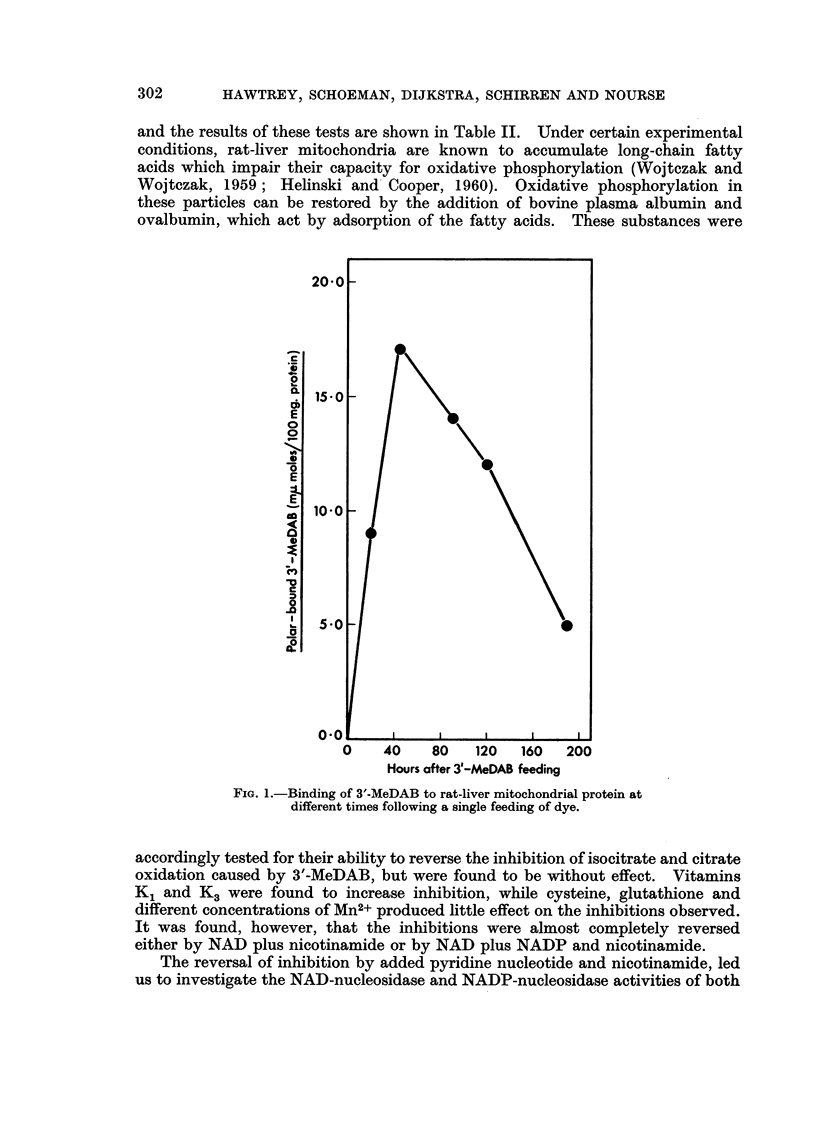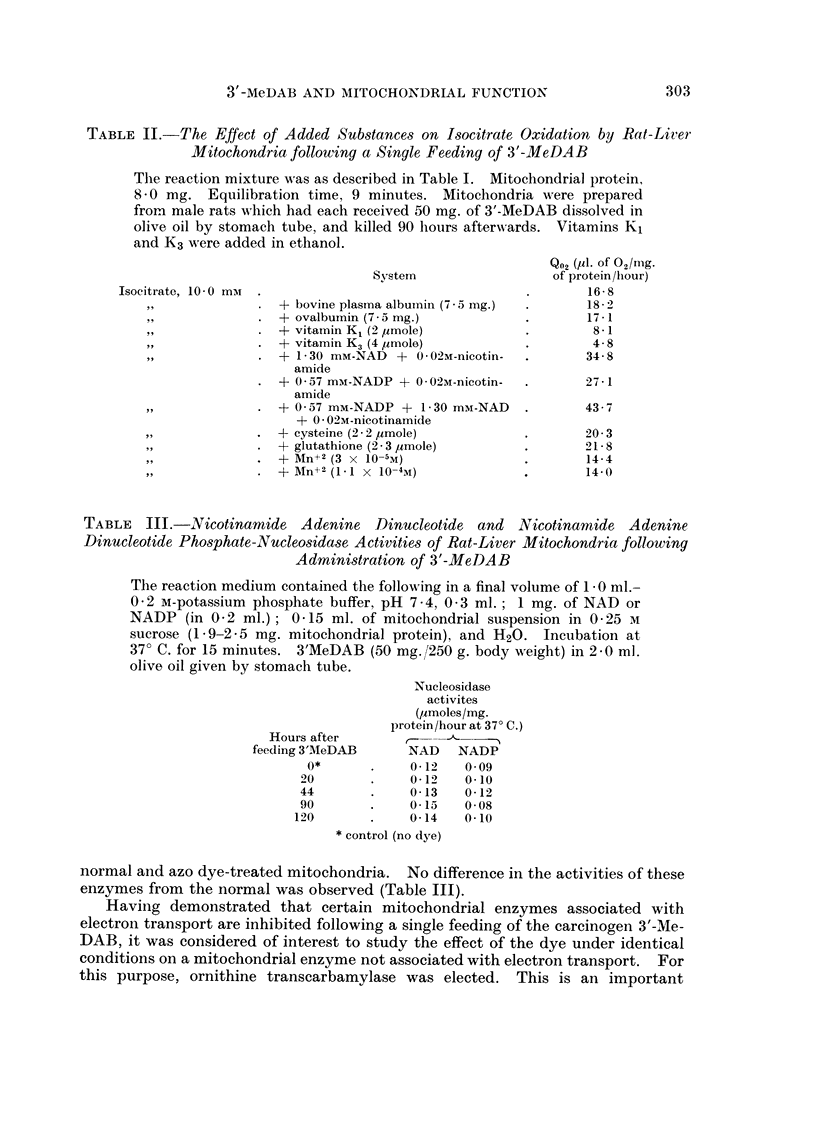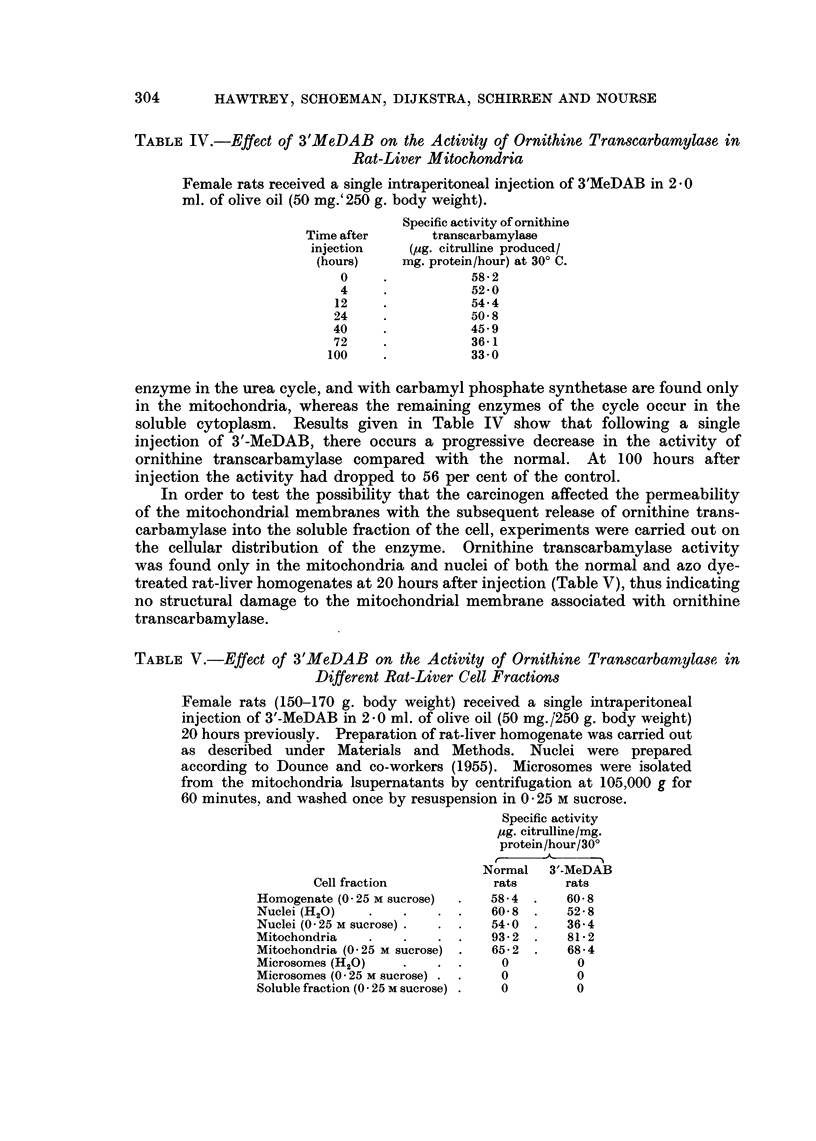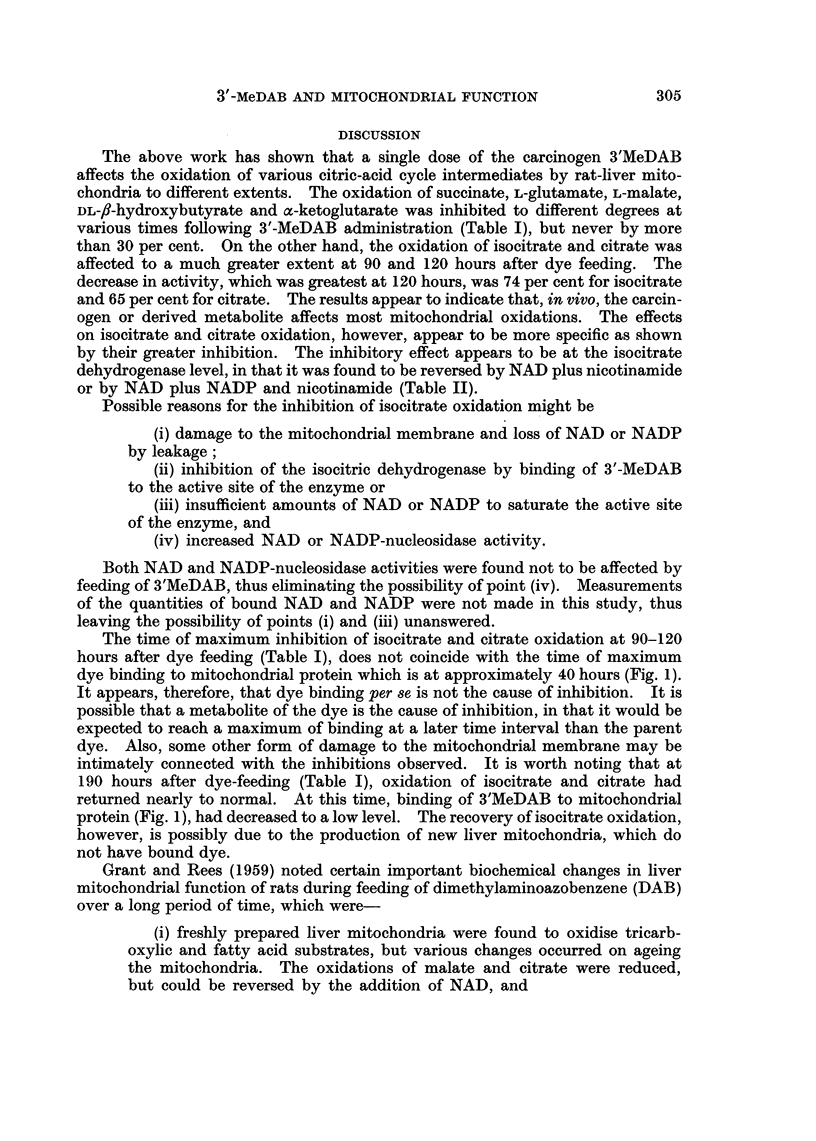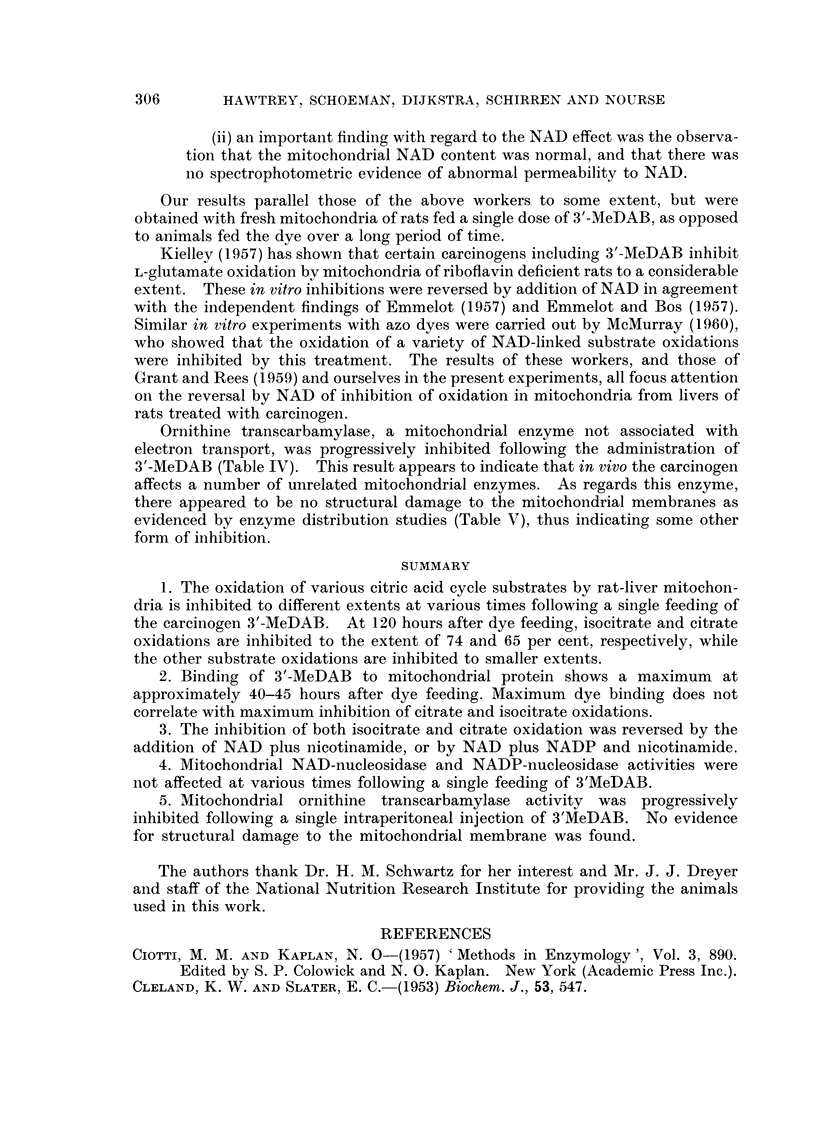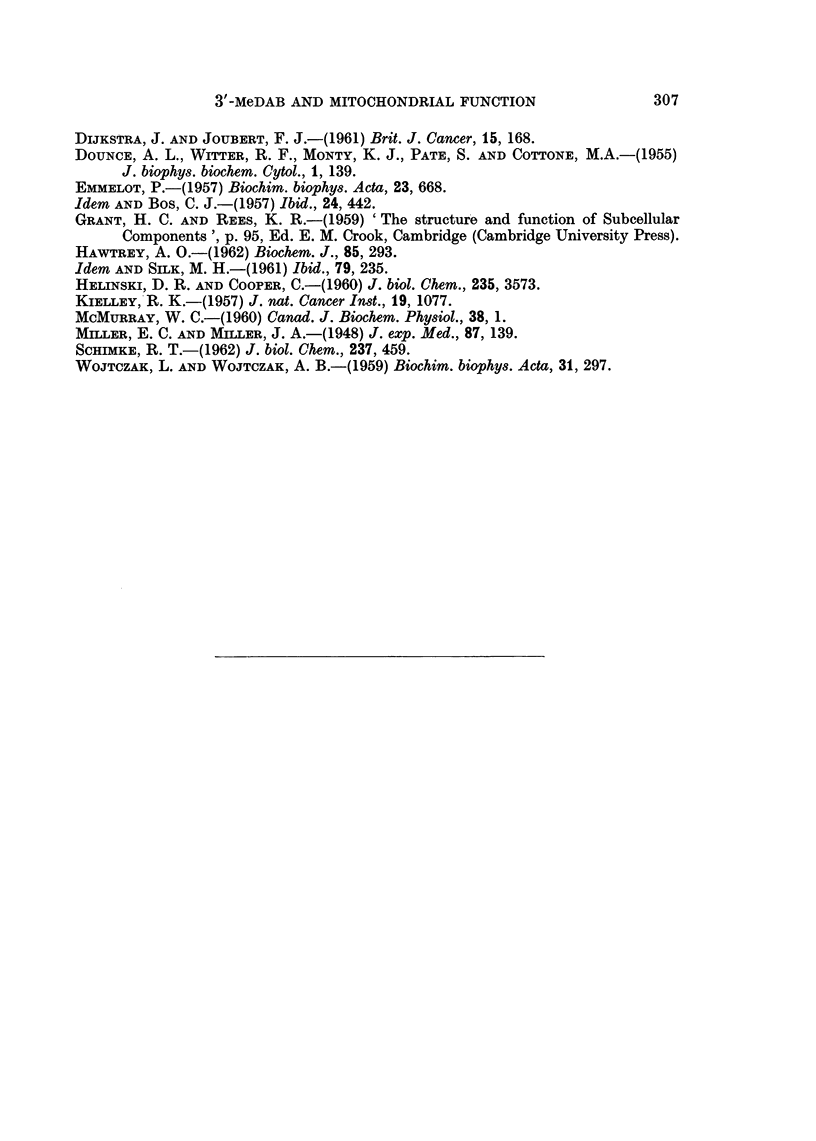# The Effect of Short-Term Feeding Experiments with 3′-Methyl-4-Dimethylaminoazobenzene on Rat-Liver Mitochondrial Function

**DOI:** 10.1038/bjc.1964.34

**Published:** 1964-06

**Authors:** A. O. Hawtrey, C. A. Schoeman, J. Dijkstra, Vera Schirren, L. Nourse


### THE EFFECT OF SHORT-TERM FEEDING EXPERIMENTS WITH 3′-METHYL-4-DIMETHYLAMINOAZOBENZENE ON RAT-LIVER MITOCHONDRIAL FUNCTION

A. O. HAWTREY, C. A. SCHOEMAN, J. DIJKSTRA, VERA SCHIRREN AND L. NOURSE

*From the National Chemical Research Laboratory and the National Nutrition Research Institute, South African Council for Scientific and Industrial Research, Pretoria, South Africa*

Received for publication March 11, 1964

We have previously described studies on isocitrate and citrate oxidation in mitochondria of normal liver and of hepatomas induced by feeding 3′-methyl-4-dimethylaminoazobenzene (3′-MeDAB) and 4-dimethylaminoazobenzene (DAB) (Hawtrey, 1962). The results of this investigation showed that normal rat-liver mitochondria possess both nicotinamide adenine dinucleotide phosphate (NADP) and nicotinamide adenine dinucleotide (NAD)-isocitrate oxidase systems, the latter demonstrable only with oxygen as terminal electron acceptor. The azo-dye-induced hepatomas, on the other hand, possess besides the normal NADP-isocitrate oxidase pathway, an NAD-dependent pathway demonstrable at the isocitric-dehydrogenase level, as well as with cytochrome C and oxygen as electron acceptors.

These differences between normal liver and azo dye-induced hepatomas suggested that it might be possible to observe early effects or changes in mitochondrial function following a single large dose of the carcinogen 3′-MeDAB. The present paper reports experiments in this direction.

### MATERIALS AND METHODS

*Reagents.*—Hexokinase (crystalline), adenosine monophosphate (AMP) and adenosine diphosphate (ADP) were supplied by General Biochemicals Inc., Chagrin Falls, Ohio, U.S.A. Cytochrome C (1 per cent w/v solution in 0·09 per cent NaCl) was obtained from the H.M. Chemical Co. of Los Angeles, California, U.S.A. Nicotinamide-adenine dinucleotide (NAD), nicotinamide-adenine dinucleotide phosphate (NADP), DL-isocitrate, and $\alpha$-ketoglutarate were obtained from Boehringer and Soehne of Germany. NAD and NADP were assayed by the cyanide addition method of Ciotti and Kaplan (1957). Vitamin $K_1$ and vitamin $K_3$ (menadione) were supplied by the Nutritional Biochemicals Corporation, Ohio, U.S.A. 3′-MeDAB (m.p. 120·4–121·2° was prepared as described by Miller and Miller (1948). All other chemicals used were of analytical reagent grade.

*Preparation of rat-liver mitochondria.*—Male albino rats (200–250 g. body weight) were fasted for 4–6 hours, and then fed a single dose of 3′-MeDAB (50 mg. in 2 ml. of olive oil/250 g. body weight) by stomach tube. The animals were killed at various times following dye feeding, and their livers removed for the preparation of mitochondria as follows—

The livers were finely cut with scissors, pulped in mortar and homogenised in 0·44 M sucrose (adjusted to pH 6·8 with dilute citric acid) according to the method of Dounce, Witter, Monty, Pate and Cottone (1955). Mitochondria were isolated from the homogenate as described by Hawtrey (1962).

*Preparation of cellular fractions for the determination of ornithine transcarbamylase activity.*—Homogenates of rat-liver were prepared as previously described in the text. Nuclei were prepared according to Dounce, Witter, Monty, Pate and Cottone (1955), and mitochondria as described above. Microsomes were isolated from the mitochondrial supernatant by centrifugation at 105,000 ***g*** for 1 hour. All cellular fractions were washed once in 0·25 M sucrose and kept at 0° C. The microsomal supernatant was taken as the soluble fraction.

*Measurement of oxygen uptake.*—This was carried out in Warburg manometers at 30° as previously described (Hawtrey and Silk, 1961).

*Spectrophotometric assays.*—Measurements with 2,6-dichlorophenolindophenol as electron acceptor were carried out in 1 cm. cuvettes using a Zeiss spectrophotometer at room temperature. Reduction of the dye was followed at 600 m$\mu$.

*Mitochondrial protein content.*—This was determined according to the biuret method of Cleland and Slater (1953), using an albumin standard.

*Determination of mitochondrial-bound dye.*—Mitochondrial suspensions in 0·25 M sucrose were treated with trichloroacetic acid to a final concentration of 5 per cent. The resulting protein precipitates were washed twice with cold 5 per cent trichloroacetic acid, and twice with 96 per cent ethanol at room temperature. Polar-bound dye in the final precipitates was estimated as described by Dijkstra and Joubert (1961).

*NAD-nucleosidase and NADP-nucleosidase activity.*—Measurements were carried out as follows—To test-tubes containing the reaction medium given in Table III was added a known amount of mitochondrial suspension, and the mixture was incubated at 37° C. under aerobic conditions with constant shaking for 15 minutes. Reactions were stopped by immersing the tubes in boiling water for 1·5 minutes, and then immediately transferring them to crushed ice. The ice-cold samples were centrifuged at 2000 r.p.m. for 10 minutes, and 0·2 ml. aliquots of the clear supernatant assayed for NAD and NADP by the cyanide addition method (Ciotti and Kaplan, 1957). Readings were taken at 325 m$\mu$.

*Ornithine transcarbamylase activity.*—Each reaction tube contained the following n a final volume of 1·0 ml.–0·05 M glycylglycine buffer (pH 8·0) ; 0·003 M L-ornithine (pH 8·0) ; 0·02 M carbamyl phosphate (lithium salt, adjusted to pH 8·0 with dilute KOH before use), and 0·05 ml. of freshly prepared rat-liver mitochondria or other cellular fraction. Reactions were started by addition of carbamyl phosphate and allowed to run for 10 minutes at 37°. Reactions were stopped by the addition of 2·5 ml. of cold 15 per cent (w/v) $HClO_4$, and denatured protein removed by centrifuging at 500 ***g*** for 10 minutes. Citrulline was estimated in the supernatant fractions by the method of Schimke (1962), using L-citrulline as standard.

### RESULTS

*Oxygen uptake by mitochondria at different times after administration of 3′-MeDAB.*—The uptake of oxygen by rat-liver mitochondria with various citric acid

TABLE I.—*Oxygen Uptake of Rat-Liver Mitochondria at Various Times following a Single Feeding of 3'-MeDAB*

Each manometric flask contained the following in a final volume of 2·0 ml. 0·045 M $K_2HPO_4$-$KH_2PO_4$ buffer (pH 7·4); 0·0075 M KCl; 0·0375 M *D*-glucose; 0·0075 M $MgCl_2$; 0·002 M ADP ($K^+$ salt, pH 7·4); 0·001 M AMP ($K^+$ salt, pH 7·4); 0·003 M EDTA (pH 7·4); 0·047 M sucrose (includes sucrose of mitochondrial suspension); 0·015 M KF; 0·02 M nicotinamide and cytochrome C(0·04 $\mu$M). Hexokinase (1–1·4 mg.) was present in all flasks. Substrate concentrations were as follows: 0·015 M succinate; 0·015 M L-glutamate; 0·01 M DL-isocitrate; 0·01 M citrate; 0·0225 M DL-$\beta$-hydroxybutyrate; 0·01 M L-malate, and 0·015 M $\alpha$-keto-glutarate. Mitochondrial suspension in 0·25 M sucrose(0·3 ml. containing 4–8 mg. mitochondrial protein). Temperature 30° C. Equilibration time 10 minutes.

| | $Q_{O_2}$ ($\mu$l. of $O_2$/mg. protein/hour at 30° C.) Time after 3'-MeDAB administration (hour) | | | | |
|---|---|---|---|---|---|
| Substrate | 0 | 44 | 90 | 120 | 192 |
| Succinate | 46·7 | 37·1 | 37·1 | 36·3 | 49·5 |
| L-Glutamate | 22·6 | 21·1 | 18·9 | 15·2 | 29·6 |
| DL-isocitrate | 48·6 | 36·9 | 16·2 | 12·6 | 35·4 |
| Citrate | 37·2 | 38·2 | 18·1 | 13·0 | 36·1 |
| DL-$\beta$-hydroxybutyrate | 21·7 | 13·2 | 14·6 | 19·0 | 15·0 |
| L-malate | 15·9 | 17·3 | 15·7 | 11·4 | 15·3 |
| $\alpha$-ketoglutarate | 18·8 | 11·7 | 12·1 | .. | 14·7 |

cycle substrates was examined at different times after a single dose of 3'-MeDAB, and the results are shown in Table I. Oxygen uptake with succinate, L-glutamate, DL-isocitrate, citrate, DL-$\beta$-hydroxybutyrate, L-malate and $\alpha$-ketoglutarate was inhibited to varying extents up to 44 hours after feeding the carcinogen. Thereafter, inhibition became more specific, and the oxidation of isocitrate and citrate was affected to the greatest extent. Thus, at 90 hours, isocitrate and citrate oxidation were inhibited 66 and 50 per cent, respectively, while at 120 hours inhibition was 74 and 65 per cent. At this time the oxidation of succinate, glutamate and malate were affected to approximately the same extent (22·4, 32·8 and 27 per cent, respectively, while $\beta$-hydroxybutyrate oxidation was inhibited to the extent of only 12·3 per cent. At 192 hours after dye administration, the oxidation of all substrates had returned to almost normal.

In order to examine the possibility that a relationship between dye binding and inhibition of isocitrate and citrate oxidation might exist, the amount of protein-bound 3'-MeDAB of the mitochondria was examined at various times following a single dose of the dye. Results in Fig. 1 show that maximum binding of 3'-MeDAB to mitochondrial protein occurs at 40–44 hours after dye adminstration. The particles do, however, show considerable dye binding at the time interval of 120 hours. At 190 hours after feeding, dye binding had decreased to a low level.

Experiments were carried out to study the nature of the inhibition of isocitrate and citrate oxidation at 90 hours after dye administration. Various co-factors and chemical substances were tested for their ability to reverse the inhibitions, and the results of these tests are shown in Table II. Under certain experimental conditions, rat-liver mitochondria are known to accumulate long-chain fatty acids which impair their capacity for oxidative phosphorylation (Wojtczak and Wojtczak, 1959; Helinski and Cooper, 1960). Oxidative phosphorylation in these particles can be restored by the addition of bovine plasma albumin and ovalbumin, which act by adsorption of the fatty acids. These substances were

FIG. 1.—Binding of 3′-MeDAB to rat-liver mitochondrial protein at different times following a single feeding of dye.

accordingly tested for their ability to reverse the inhibition of isocitrate and citrate oxidation caused by 3′-MeDAB, but were found to be without effect. Vitamins $K_1$ and $K_3$ were found to increase inhibition, while cysteine, glutathione and different concentrations of $Mn^{2+}$ produced little effect on the inhibitions observed. It was found, however, that the inhibitions were almost completely reversed either by NAD plus nicotinamide or by NAD plus NADP and nicotinamide.

The reversal of inhibition by added pyridine nucleotide and nicotinamide, led us to investigate the NAD-nucleosidase and NADP-nucleosidase activities of both

TABLE II.—*The Effect of Added Substances on Isocitrate Oxidation by Rat-Liver Mitochondria following a Single Feeding of 3′-MeDAB*

The reaction mixture was as described in Table I. Mitochondrial protein, 8·0 mg. Equilibration time, 9 minutes. Mitochondria were prepared from male rats which had each received 50 mg. of 3′-MeDAB dissolved in olive oil by stomach tube, and killed 90 hours afterwards. Vitamins $K_1$ and $K_3$ were added in ethanol.

| | System | $Q_{O_2}$ (μl. of $O_2$/mg. of protein/hour) |
|---|---|---|
| Isocitrate, 10·0 mM | | 16·8 |
| ,, | + bovine plasma albumin (7·5 mg.) | 18·2 |
| ,, | + ovalbumin (7·5 mg.) | 17·1 |
| ,, | + vitamin $K_1$ (2 μmole) | 8·1 |
| ,, | + vitamin $K_3$ (4 μmole) | 4·8 |
| ,, | + 1·30 mM-NAD + 0·02M-nicotinamide | 34·8 |
| | + 0·57 mM-NADP + 0·02M-nicotinamide | 27·1 |
| ,, | + 0·57 mM-NADP + 1·30 mM-NAD + 0·02M-nicotinamide | 43·7 |
| ,, | + cysteine (2·2 μmole) | 20·3 |
| ,, | + glutathione (2·3 μmole) | 21·8 |
| ,, | + $Mn^{+2}$ (3 × $10^{-5}$M) | 14·4 |
| ,, | + $Mn^{+2}$ (1·1 × $10^{-4}$M) | 14·0 |

TABLE III.—*Nicotinamide Adenine Dinucleotide and Nicotinamide Adenine Dinucleotide Phosphate-Nucleosidase Activities of Rat-Liver Mitochondria following Administration of 3′-MeDAB*

The reaction medium contained the following in a final volume of 1·0 ml.– 0·2 M-potassium phosphate buffer, pH 7·4, 0·3 ml.; 1 mg. of NAD or NADP (in 0·2 ml.); 0·15 ml. of mitochondrial suspension in 0·25 M sucrose (1·9–2·5 mg. mitochondrial protein), and $H_2O$. Incubation at 37° C. for 15 minutes. 3′MeDAB (50 mg./250 g. body weight) in 2·0 ml. olive oil given by stomach tube.

| Hours after feeding 3′MeDAB | Nucleosidase activites (μmoles/mg. protein/hour at 37° C.) | |
|---|---|---|
| | NAD | NADP |
| 0* | 0·12 | 0·09 |
| 20 | 0·12 | 0·10 |
| 44 | 0·13 | 0·12 |
| 90 | 0·15 | 0·08 |
| 120 | 0·14 | 0·10 |

* control (no dye)

normal and azo dye-treated mitochondria. No difference in the activities of these enzymes from the normal was observed (Table III).

Having demonstrated that certain mitochondrial enzymes associated with electron transport are inhibited following a single feeding of the carcinogen 3′-Me-DAB, it was considered of interest to study the effect of the dye under identical conditions on a mitochondrial enzyme not associated with electron transport. For this purpose, ornithine transcarbamylase was elected. This is an important

TABLE IV.—*Effect of 3′MeDAB on the Activity of Ornithine Transcarbamylase in Rat-Liver Mitochondria*

Female rats received a single intraperitoneal injection of 3′MeDAB in 2·0 ml. of olive oil (50 mg.‘250 g. body weight).

| Time after injection (hours) | Specific activity of ornithine transcarbamylase (μg. citrulline produced/ mg. protein/hour) at 30° C. |
|---|---|
| 0 | 58·2 |
| 4 | 52·0 |
| 12 | 54·4 |
| 24 | 50·8 |
| 40 | 45·9 |
| 72 | 36·1 |
| 100 | 33·0 |

enzyme in the urea cycle, and with carbamyl phosphate synthetase are found only in the mitochondria, whereas the remaining enzymes of the cycle occur in the soluble cytoplasm. Results given in Table IV show that following a single injection of 3′-MeDAB, there occurs a progressive decrease in the activity of ornithine transcarbamylase compared with the normal. At 100 hours after injection the activity had dropped to 56 per cent of the control.

In order to test the possibility that the carcinogen affected the permeability of the mitochondrial membranes with the subsequent release of ornithine transcarbamylase into the soluble fraction of the cell, experiments were carried out on the cellular distribution of the enzyme. Ornithine transcarbamylase activity was found only in the mitochondria and nuclei of both the normal and azo dye-treated rat-liver homogenates at 20 hours after injection (Table V), thus indicating no structural damage to the mitochondrial membrane associated with ornithine transcarbamylase.

TABLE V.—*Effect of 3′MeDAB on the Activity of Ornithine Transcarbamylase in Different Rat-Liver Cell Fractions*

Female rats (150–170 g. body weight) received a single intraperitoneal injection of 3′-MeDAB in 2·0 ml. of olive oil (50 mg./250 g. body weight) 20 hours previously. Preparation of rat-liver homogenate was carried out as described under Materials and Methods. Nuclei were prepared according to Dounce and co-workers (1955). Microsomes were isolated from the mitochondria lsupernatants by centrifugation at 105,000 *g* for 60 minutes, and washed once by resuspension in 0·25 M sucrose.

| Cell fraction | Specific activity μg. citrulline/mg. protein/hour/30° — Normal rats | 3′-MeDAB rats |
|---|---|---|
| Homogenate (0·25 M sucrose) | 58·4 | 60·8 |
| Nuclei ($H_2O$) | 60·8 | 52·8 |
| Nuclei (0·25 M sucrose) | 54·0 | 36·4 |
| Mitochondria | 93·2 | 81·2 |
| Mitochondria (0·25 M sucrose) | 65·2 | 68·4 |
| Microsomes ($H_2O$) | 0 | 0 |
| Microsomes (0·25 M sucrose) | 0 | 0 |
| Soluble fraction (0·25 M sucrose) | 0 | 0 |

### DISCUSSION

The above work has shown that a single dose of the carcinogen 3′MeDAB affects the oxidation of various citric-acid cycle intermediates by rat-liver mitochondria to different extents. The oxidation of succinate, L-glutamate, L-malate, DL-$\beta$-hydroxybutyrate and $\alpha$-ketoglutarate was inhibited to different degrees at various times following 3′-MeDAB administration (Table I), but never by more than 30 per cent. On the other hand, the oxidation of isocitrate and citrate was affected to a much greater extent at 90 and 120 hours after dye feeding. The decrease in activity, which was greatest at 120 hours, was 74 per cent for isocitrate and 65 per cent for citrate. The results appear to indicate that, *in vivo*, the carcinogen or derived metabolite affects most mitochondrial oxidations. The effects on isocitrate and citrate oxidation, however, appear to be more specific as shown by their greater inhibition. The inhibitory effect appears to be at the isocitrate dehydrogenase level, in that it was found to be reversed by NAD plus nicotinamide or by NAD plus NADP and nicotinamide (Table II).

Possible reasons for the inhibition of isocitrate oxidation might be

(i) damage to the mitochondrial membrane and loss of NAD or NADP by leakage ;

(ii) inhibition of the isocitric dehydrogenase by binding of 3′-MeDAB to the active site of the enzyme or

(iii) insufficient amounts of NAD or NADP to saturate the active site of the enzyme, and

(iv) increased NAD or NADP-nucleosidase activity.

Both NAD and NADP-nucleosidase activities were found not to be affected by feeding of 3′MeDAB, thus eliminating the possibility of point (iv). Measurements of the quantities of bound NAD and NADP were not made in this study, thus leaving the possibility of points (i) and (iii) unanswered.

The time of maximum inhibition of isocitrate and citrate oxidation at 90–120 hours after dye feeding (Table I), does not coincide with the time of maximum dye binding to mitochondrial protein which is at approximately 40 hours (Fig. 1). It appears, therefore, that dye binding *per se* is not the cause of inhibition. It is possible that a metabolite of the dye is the cause of inhibition, in that it would be expected to reach a maximum of binding at a later time interval than the parent dye. Also, some other form of damage to the mitochondrial membrane may be intimately connected with the inhibitions observed. It is worth noting that at 190 hours after dye-feeding (Table I), oxidation of isocitrate and citrate had returned nearly to normal. At this time, binding of 3′MeDAB to mitochondrial protein (Fig. 1), had decreased to a low level. The recovery of isocitrate oxidation, however, is possibly due to the production of new liver mitochondria, which do not have bound dye.

Grant and Rees (1959) noted certain important biochemical changes in liver mitochondrial function of rats during feeding of dimethylaminoazobenzene (DAB) over a long period of time, which were—

(i) freshly prepared liver mitochondria were found to oxidise tricarboxylic and fatty acid substrates, but various changes occurred on ageing the mitochondria. The oxidations of malate and citrate were reduced, but could be reversed by the addition of NAD, and

(ii) an important finding with regard to the NAD effect was the observation that the mitochondrial NAD content was normal, and that there was no spectrophotometric evidence of abnormal permeability to NAD.

Our results parallel those of the above workers to some extent, but were obtained with fresh mitochondria of rats fed a single dose of 3′-MeDAB, as opposed to animals fed the dye over a long period of time.

Kielley (1957) has shown that certain carcinogens including 3′-MeDAB inhibit L-glutamate oxidation by mitochondria of riboflavin deficient rats to a considerable extent. These *in vitro* inhibitions were reversed by addition of NAD in agreement with the independent findings of Emmelot (1957) and Emmelot and Bos (1957). Similar *in vitro* experiments with azo dyes were carried out by McMurray (1960), who showed that the oxidation of a variety of NAD-linked substrate oxidations were inhibited by this treatment. The results of these workers, and those of Grant and Rees (1959) and ourselves in the present experiments, all focus attention on the reversal by NAD of inhibition of oxidation in mitochondria from livers of rats treated with carcinogen.

Ornithine transcarbamylase, a mitochondrial enzyme not associated with electron transport, was progressively inhibited following the administration of 3′-MeDAB (Table IV). This result appears to indicate that *in vivo* the carcinogen affects a number of unrelated mitochondrial enzymes. As regards this enzyme, there appeared to be no structural damage to the mitochondrial membranes as evidenced by enzyme distribution studies (Table V), thus indicating some other form of inhibition.

SUMMARY

1. The oxidation of various citric acid cycle substrates by rat-liver mitochondria is inhibited to different extents at various times following a single feeding of the carcinogen 3′-MeDAB. At 120 hours after dye feeding, isocitrate and citrate oxidations are inhibited to the extent of 74 and 65 per cent, respectively, while the other substrate oxidations are inhibited to smaller extents.

2. Binding of 3′-MeDAB to mitochondrial protein shows a maximum at approximately 40–45 hours after dye feeding. Maximum dye binding does not correlate with maximum inhibition of citrate and isocitrate oxidations.

3. The inhibition of both isocitrate and citrate oxidation was reversed by the addition of NAD plus nicotinamide, or by NAD plus NADP and nicotinamide.

4. Mitochondrial NAD-nucleosidase and NADP-nucleosidase activities were not affected at various times following a single feeding of 3′MeDAB.

5. Mitochondrial ornithine transcarbamylase activity was progressively inhibited following a single intraperitoneal injection of 3′MeDAB. No evidence for structural damage to the mitochondrial membrane was found.

The authors thank Dr. H. M. Schwartz for her interest and Mr. J. J. Dreyer and staff of the National Nutrition Research Institute for providing the animals used in this work.

### REFERENCES

CIOTTI, M. M. AND KAPLAN, N. O—(1957) 'Methods in Enzymology', Vol. 3, 890. Edited by S. P. Colowick and N. O. Kaplan. New York (Academic Press Inc.).

CLELAND, K. W. AND SLATER, E. C.—(1953) *Biochem. J.*, **53**, 547.

DIJKSTRA, J. AND JOUBERT, F. J.—(1961) *Brit. J. Cancer*, **15**, 168.
DOUNCE, A. L., WITTER, R. F., MONTY, K. J., PATE, S. AND COTTONE, M.A.—(1955) *J. biophys. biochem. Cytol.*, **1**, 139.
EMMELOT, P.—(1957) *Biochim. biophys. Acta*, **23**, 668.
*Idem* AND BOS, C. J.—(1957) *Ibid.*, **24**, 442.
GRANT, H. C. AND REES, K. R.—(1959) 'The structure and function of Subcellular Components', p. 95, Ed. E. M. Crook, Cambridge (Cambridge University Press).
HAWTREY, A. O.—(1962) *Biochem. J.*, **85**, 293.
*Idem* AND SILK, M. H.—(1961) *Ibid.*, **79**, 235.
HELINSKI, D. R. AND COOPER, C.—(1960) *J. biol. Chem.*, **235**, 3573.
KIELLEY, R. K.—(1957) *J. nat. Cancer Inst.*, **19**, 1077.
MCMURRAY, W. C.—(1960) *Canad. J. Biochem. Physiol.*, **38**, 1.
MILLER, E. C. AND MILLER, J. A.—(1948) *J. exp. Med.*, **87**, 139.
SCHIMKE, R. T.—(1962) *J. biol. Chem.*, **237**, 459.
WOJTCZAK, L. AND WOJTCZAK, A. B.—(1959) *Biochim. biophys. Acta*, **31**, 297.